# Juvenile/Adolescent Idiopatic Scoliosis and Rapid Palatal Expansion. A Pilot Study

**DOI:** 10.3390/children8050362

**Published:** 2021-04-30

**Authors:** Maria Grazia Piancino, Francesco MacDonald, Ivana Laponte, Rosangela Cannavale, Vito Crincoli, Paola Dalmasso

**Affiliations:** 1 Department of Surgical Sciences, Dental School C.I.R., Division of Orthodontics, University of Turin, 10126 Turin, Italy; 2 Spine Care and Deformity Division, Hospital Company Maria Adelaide, 10126 Turin, Italy; Francesco.macdonald@libero.it; 3 Private Practice, 20831 Milan, Italy; laponte@libero.it; 4 Department of Surgical Sciences-Orthodontic Division, PhD School, University of Turin, 10126 Turin, Italy; cannavale@alice.it; 5 Department of Basic Medical Sciences, Neurosciences and Sensory Organs, Division of Complex Operating Unit of Dentistry, University of Bari, 70121 Bari, Italy; vito.crincoli@uniba.it; 6 Department of Public Health and Pediatrics, School of Medicine, University of Turin, 10126 Turin, Italy; paola.dalmasso@unito.it

**Keywords:** scoliosis, orthopedics, malocclusion, orthodontics

## Abstract

The question of whether orthodontic therapy by means of rapid palatal expansion (RPE) affects the spine during development is important in clinical practice. RPE is an expansive, fixed therapy conducted with heavy forces to separate the midpalatal suture at a rate of 0.2–0.5 mm/day. The aim of the study was to evaluate the influence of RPE on the curves of the spine of juvenile/adolescent idiopathic scoliosis patients. Eighteen patients under orthopedic supervision for juvenile/adolescent idiopathic scoliosis and independently treated with RPE for orthodontic reasons were included in the study: Group A, 10 subjects (10.4 ± 1.3 years), first spinal radiograph before the application of the RPE, second one during the orthodontic therapy with RPE; Group B, 8 patients (11.3 ± 1.6 years), first radiograph during the use of RPE second one after the removal. Group A showed a significant worsening of the Cobb angle (*p* ≤ 0.005) at the second radiograph after RPE. Group B showed a significant improvement of the Cobb angle (*p* = 0.01) at the second radiograph after removal of RPE. Based on the results, the use of RPE during adolescence might influence the spinal curves of patients with idiopathic scoliosis.

## 1. Introduction

The relationship between occlusion, craniofacial morphology and spine posture is still a debated topic: the results regarding the correlation between occlusion and posture are not in agreement. Around half of the manuscripts show an association, the other half do not confirm any association [1,2]. This might be due to the complexity of the topic and to inhomogeneous studies.

Among postural pathologies, idiopathic scoliosis is a developmental deformation of the spine of an unknown etiology. It may affect the stomatognathic system, but the reverse may also be true, i.e., some malocclusions are often diagnosed in subjects with scoliosis. The frequent presence of some malocclusions in patients with scoliosis has been shown, especially asymmetrical malocclusions as crossbites, bilateral class II or more often asymmetrical class II with deviation of the median line, and open bites [3,4,5,6]. Some studies conclude suggesting a multidisciplinary approach between orthodontists and orthopedics before planning the orthodontic treatment [7]. However, a recent systematic review aiming to deepen the association between spinal deformities and malocclusions [8] highlighted the medium-low quality of the manuscripts; the authors underlined that two interesting animal studies showed a role of an induced malocclusion on scoliotic irreversible degeneration and three studies suggested an increased prevalence of occlusal dysfunction in patients with known spinal deformity. Nevertheless, based on the results of the review, the conclusion is that at the current state of knowledge no evidence of causal relationship between occlusal alterations and spinal deformity in both directions could be supported, as well as a therapeutic effect. It seems that this important topic for specific populations, especially in adulthood when chronic back pain arises, is still difficult to investigate [9,10,11].

Rapid palatal expansion (RPE) is an expansive, fixed therapy conducted with heavy forces able to separate the midpalatal suture at a rate of 0.2–0.5 mm/d. This approach takes advantage of the patency of the craniofacial sutures in children, especially the midpalatal suture [12]. It is widely used all over the world during the developmental period and before fusion of the palatal suture; it is the preferred option of orthodontic treatment in order to avoid subsequent palatal transversal discrepancy and the need for surgical treatment by methods like corticotomy, SARPE or segmental maxillary osteotomy at maturation. When using RPE in growing children, the knowledge of benefits and side effects, which are still minimally considered with respect to the importance of the therapy, is needed. The side effects reported concern dental aspects, especially periodontal damage in the molar and premolar region, buccal tipping of the anchor teeth and alveolar processes which may lead to posterior rotation of the mandible, open bite, an increase in facial vertical dimension [13,14], and external root resorption [15], but also medical aspects such as transient bacteremia after expander removal [16], bleedings, diplopia [17] and speech problems [18]. The outcomes and the possible side effects should be considered before using this type of therapy [19].

Cranial and postural systems are complex and the attempts to show the relationship between malocclusions and posture have failed, even with clinical trials. It may be that generalized methods are not suitable for these studies and a more precise multidisciplinary approach is required. The only one randomized clinical study regarding the influence of Rapid Palatal Expansion (RPE) on spine posture [20] aimed to evaluate if early RPE could negatively affect the postural parameters. Unfortunately, the quality of the study was low [8], meaning there was a high risk of biases. The instrument used to assess the spine posture was the rastrenography, where unreliability has been highlighted in the literature [8]. To date, the gold standard for identifying and monitoring scoliosis is the standing anteroposterior (AP) and lateral scoliosis X-ray films, with systematic radiographic imaging performed throughout the individual’s course of treatment. One of the difficulties of this research is the fact that a multidisciplinary approach between orthodontists and orthopedics is essential, but not easy to realize in everyday practice.

The question of whether orthodontic therapy by means of RPE may affect the spine during development is of importance in clinical practice. Despite the importance of the topic, there are not many studies in the literature, probably due to the difficulty of planning this research since radiographic imaging of the spine is of orthopedic expertise, while the cranial is of orthognathic expertise. To answer the question, this pilot study was planned based on a multidisciplinary collaboration between orthopedics and orthodontists.

The aim of the study was to evaluate the influence of RPE used for orthodontic reasons on the spine curves of patients with juvenile/adolescent idiopathic scoliosis, by means of Cobb angle measurements and Risser staging.

## 2. Materials and Methods

Eighteen patients following orthopedic treatment for juvenile and adolescent idiopathic scoliosis in private practice and who were independently treated with RPE for orthodontic reasons to expand the palate were selected out of 380 patients and were included in this retrospective study which lasted three years from January 2017 to January 2020. The inclusion criteria were juvenile/adolescent idiopathic scoliosis under orthopedic treatment or orthopedic control, simultaneous treatment with RPE, at least two spinal radiographs before and after the application of the orthodontic device. Exclusion criteria were juvenile/adolescent idiopathic scoliosis without RPE appliance or with a different appliance or without any appliance, and one spinal radiograph only.

The totality of patients underwent the spinal radiographs for orthopedic reasons and the Cobb angle was measured on all the spinal radiographs as well as the staging of Risser [11] by two blinded skilled operators; the gap between the measurements was comprised within 1°–2° and the mean value was considered for the statistical analysis. The US Risser system was used to highlight the intermediate values, and the Risser data were expressed in percent. The reference diagram in percent is shown in Table 1.

The spinal radiographs taken for orthopedic reasons without the orthodontic appliance (group A) were compared to that of the same patients after the application of the appliance; the X-rays of a second group (B), also taken for orthopedic reasons but with the appliance in the mouth, were compared with those of the same patients taken after the removal.

Group A was made of patients under orthopedic supervision and to which the orthodontist independently applied the RPE device for orthodontic reasons; after this, the orthopedist observed an inexplicable worsening of the spinal curves, proven by the spinal radiographs. Group B was made of patients who took out the RPE, allowing the orthopedist to observe a dramatic improvement of the scoliosis which was out of the expected evolution of the curves.

All patients underwent rapid palatal expansion which is a standardized orthodontic therapy that provides the application of a fixed appliance anchored at the upper teeth (molars, premolars and canine), with thick bars from one side to the other of the dental arches and a screw in the middle of the palate to be activated for 2 to 6 weeks (2 turns per day, 0.25 mm per turn), followed by a retention period [21].

The patients were divided into two groups:

Group A, 10 subjects (age: mean ± SD (Standard Deviation): 10.4 ± 1.3 years) underwent the first spinal radiograph before the application of the device and the second during orthodontic therapy with RPE.

Group B, 8 patients (age: mean ± SD:11.3 ± 1.6 years) underwent the first spinal radiograph during therapy with RPE and the second after the removal of the orthodontic device.

All patients were under orthopedic supervision and underwent full- or part-time treatment with original Cheneau brace and motor activity adapted to the brace after the removal of the orthodontic device. The expected result after the use of the Cheneau brace is a mean improvement of 20% of the scoliotic curves.

### Statistical Analysis

Data were expressed as mean ± SD and median (IQR). The statistical distribution of the quantitative measures was tested by Shapiro–Wilk test and showed non-Gaussian distribution. Comparisons within groups were performed by Wilcoxon signed-rank test. Cobb and Risser differences between first and second evaluation among the two groups were assessed using a two-sample Wilcoxon rank-sum (Mann–Whitney) test. All the tests were two-tailed and the level of significance was set at 5%.

## 3. Results

**Figure 1:**
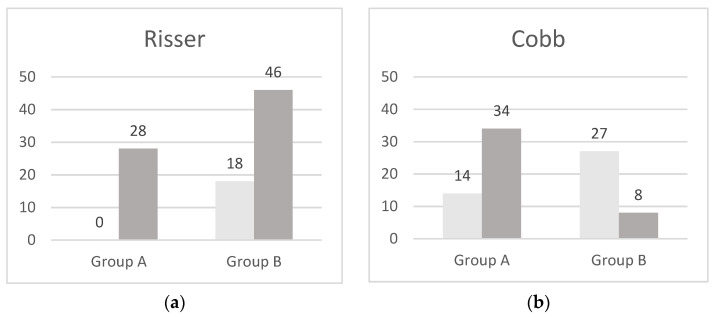
Histograms of the Risser value (**a**) and Cobb angle (**b**) in Group A (comparison before/after RPE, Risser *p* ≤ 0.007, Cobb *p* ≤ 0.005) and in Group B (comparison with RPE and after removal, Risser *p* ≤ 0.01, Cobb *p* ≤ 0.01).

Group A: The results reported in Table 2 show a significant difference for Risser (*p* ≤ 0.007) and a worsening of the Cobb angle (*p* ≤ 0.005) between the first (without the appliance) and the second spinal radiograph (after the orthodontic appliance) (Figure 1).

Group B: The results reported in Table 2 show a significant difference for Risser (*p* ≤ 0.01) and an improvement of the Cobb angle (*p* ≤ 0.01) between the first (with the orthodontic appliance) and the second spinal radiograph (after the removal) (Figure 1).

## 4. Discussion

This pilot study was aimed at highlighting the influence of RPE used for orthodontic reasons on the spinal curves of patients with juvenile/adolescent idiopathic scoliosis by means of spinal radiographs. To our knowledge, this is the first study based on spinal radiographs with and without the RPE appliances.

Both patient groups showed a high significant difference between the condition with and without the RPE appliance. Group A showed a significant worsening of the Cobb angle (*p* = 0.005) after RPE; Group B showed a significant improvement of the Cobb angle (*p* = 0.01) after the removal of the RPE appliance.

The uniqueness of this study is due to the spinal radiographs, the orthopedic diagnosis and the evaluation of scoliosis before and after the use of the RPE appliance. This cannot be done by orthodontists alone, even if they are the experts in the field, as they cannot diagnose nor treat patients from an orthopedic point of view. On the other hand, orthopedists do not know the mechanical actions of orthodontic appliances that are managed by a niche specialist. These cases have been collected after a close collaboration between orthodontists and orthopedics in an effort to understand the complex relationships between the cranial and postural systems.

RPE is a fixed therapy anchored to the upper dental arch. The forces are applied to the anterior (canine) and posterior (molars and premolars) teeth. The entity of the mean mechanical activation is 10 mm (mm) but the mean inter-canine distance after RPE results in an overall gain of 2.91 mm (a gain of 3.73 mm and a relapse of −0.81 mm) and the mean intermolar distance results in an overall gain of 4.38 mm (a gain of 4.85 mm and a relapse of −0.47) [22]. Even if the mechanical action is symmetrical, this technique is used in a large majority of patients with important asymmetrical skulls and malocclusions, i.e., crossbites and asymmetrical molar class II.

**Figure 2:**
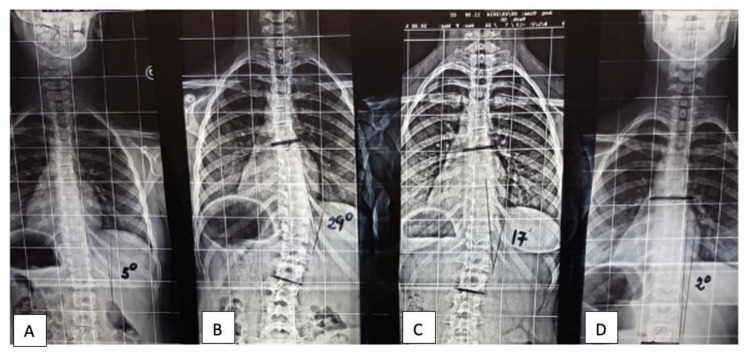
X-ray of the spine of a patient before and after rapid expansion of the palatal suture (RPE). (**A**) Cobb 5° Risser 0%. Without appliance. (**B**) Cobb 29° Risser 20%. 6 months after RPE device application. (**C**) Cobb 17° Risser 40%. 6 months after the removal of RPE device. (**D**) Cobb 2° Risser 60%. 12 months after the removal of RPE device.

From an orthopedic point of view, the main consideration is the fact that the worsening of the Cobb angle after the application of the appliance and its improvement after the removal is totally in disagreement with the known evolution of the scoliotic curves in pre-adolescents and adolescent patients (Figure 2). We know that infantile scoliosis from 0 to 3 years of age could be progressive or resolving; juvenile scoliosis (from 3 years of age through pubertal spurt) worsens 3/4° per year; adolescent scoliosis worsens 1° per month from puberty to the end of growth [21,23,24]. We also know that the best therapy is not able to completely control the worsening of the curve, meaning that an aggravation is expected at the end of growth anyway. For this reason, a spontaneous worsening of the Cobb angle of patients with adolescent idiopathic scoliosis was expected in Group B. Unexpectedly, the mean Cobb angle of Group B after the appliance removal dramatically improved from (mean ± SD) 28.2 ± 6.9° to 6.2 ± 11.0° (*p* ≤ 0.005). To this end, it is reasonable to think that there may have been an additional external cause in the etiopathogenesis of these curves.

Moreover, the serious worsening of the curves of Group A from (mean ± SD) 13.9 ± 16.1° to 34.2 ± 20.5° after the application of the appliance is surprising and worrying. An important evolution of a scoliotic curve is out of the known statistical forecast. An external factor could have influenced the exacerbation of the scoliotic curves of Group A; as well, the improvement of Group B could be considered the opposite proof of the influence of an external cause on the scoliotic curves. The possible external cause common to all cases of this pilot study is the RPE fixed appliance cemented on the teeth.

Of course, a higher number of cases is necessary to better understand the phenomena, but interestingly, all the cases of both groups showed the same tendency unanimously, with all worsening in Group A and all improving in Group B. These results should make us reflect on the possible influence of this mechanical appliance on the posture of the soma. Fortunately, after the removal of the appliance a significant improvement of the curves was observed. At the present state of knowledge, we do not know to what extent and after how long the curves could become structured. To this end, it could be worth underlining that the relationship between the masticatory muscles and the neck muscles is known after a long time [25,26]. It is likely that the muscles of neck and spine may react to the asymmetries of the skull and mandible in the new environment created by the orthodontic device that stiffens the upper arch, giving rise to new pre-contacts starting or worsening the scoliotic curves. The muscular involvement could explain the dramatic improvement of the curves after taking the appliance out of the mouth. This is possibly due to the young age of patients who are able to quickly compensate, but the risk that the curve becomes structured with irreversible bone damages is worrying. Indeed, some animal studies showed irreversible scoliotic degeneration after malocclusions [27]. This will be an important future direction of the research.

The limitation of the study is the limited number of cases due to the strict inclusion criteria and the retrospective planning. Nevertheless, we thought it worthwhile to share this preliminary data for their significance from the clinical point of view. A future direction of this research to improve our knowledge in the field could be the selection of a group of children suffering from juvenile/adolescent idiopathic scoliosis treated with different orthodontic therapies.

In agreement with other studies [7] and based on the results of this study, it would be advisable and conservative to possibly deeply evaluate the use of RPE in predisposed subjects and in patients affected by juvenile/adolescent idiopathic scoliosis in favor of gnathological therapies that are respectful of the physiology of the system. Gnathological therapies in orthodontics are therapies characterized by the protection of the upper from the lower teeth, the self-repositioning of the mandible in the three planes of space in the best-balanced position and the use of intermittent self-regulated forces during the orthodontic movement; this is nowadays easily possible and will be the next direction of this research [28,29,30].

## 5. Conclusions

Based on the results of this study, we can conclude that the use of RPE devices in patients with juvenile/idiopathic scoliosis might have an influence on the spinal curves. The results fully showed a worsening of the spinal curves after RPE and an improvement after removal. Being that idiopathic scoliosis is a serious disabling disease, it could be advisable in children with juvenile/adolescent idiopathic scoliosis to organize an orthopedic consultation before planning orthodontic therapy. Further investigations are required to establish a deeper understanding of the human body as a whole to evolve toward a true multidisciplinary approach.

## Figures and Tables

**Table 1:** Reference diagram in percentages to illustrate the intermediate Risser values.

Risser 0	0–10%
Risser 1	20–30%
Risser 2	40–50%
Risser 3	60–70%
Risser 4	80–90%
Risser 5	100%

**Table 2:** Mean ± standard deviation of age, Risser value, Cobb angle and median (IQR) of Group A and B.

	**Group A (*n* = 10)**		
	Before RPE	With RPE	*p* *
Age	10.4 ± 1.3	12.8 ± 1.5	
Risser (mean ± sd) Median (IQR)	0	28 ± 23.9 20 (10–40)	0.007
Cobb (mean ± sd) Median (IQR)	13.9 ± 16.1 6.5 (0–27)	34 ± 20.5 35.5 (12–45)	0.005
	**Group B (*n* = 8)**		
	With RPE	After removal of RPE	*p* *
Age	11.3 ± 1.7	13.6 ± 1.0	
Risser(mean ± sd) Median (IQR)	17.5 ± 16.7 15 (5–25)	46.3 ± 15.3 45 (35–58)	0.01
Cobb (mean ± sd) Median (IQR)	27.4 ± 7.6 30 (25–32)	7.8 ± 11.9 1 (0–16)	0.01

RPE: Rapid Palatal Expansion; * level of significance *p* < 0.05.

## Data Availability

Data available on request from the corresponding author.
